# On the Pathology of the Pancreas

**Published:** 1855-09

**Authors:** 


*On the Pathology of the Pancreas.* By Dr. Eisenmann.—The interesting experiments of M. Cl. Bernard upon the uses of the pancreatic juice, have led the author of this article to consider, whether or not the results, obtained by that physiologist, are capable of being applied to the diagnosis of disease in this organ.

If the pancreatic juice assists the digestion, and consequently the absorption of fatty matters, the conclusion is, that when the functions of that gland are destroyed by disease, fat should pass with the dejections, and be found in them in an unaltered condition. It is interesting to observe how nearly science has anticipated what the observations at the bedside of patients laboring under such disease have confirmed. The author has united the facts observed by others with those presented in a case of his own.

Of seven cases adduced, six terminated in death; there had been abundant fatty evacuations, and *post mortem* examination showed the existence of induration, or other alteration in the pancreas. In the seventh case, quoted from Lussana, the patient recovered. The principal symptoms in this case were copious salivation, a sense of weight in the epigastrium not increased upon pressure, eructations, flatulence, coldness of the surface, and a small and slow pulse. The expression indicated abdominal disease. Constipation was present, but the fæces contained yellowish particles resembling fatty concretions, and which were in fact composed of fatty materials. These occurred in increased quantity after purgatives. M. Eisenmann regards Lussana as the first who applied to pathology the discovery of Bernard. In the instance observed by himself, the author had diagnosed pancreatic disease, but had not made out the presence of fat in the evacuations. This circumstance induced him to suppose that the pancreatic juice did not exclusively produce the absorption of fatty matters, but was assisted in this action by the bile. Budge and Wistinghausen have made experiments which countenance this idea, and the author recalls a case cited by Pearson, of a woman who evacuated daily 3 oz. of a fatty substance, and did not present, upon *post mortem* examination, any trace of pencreatic disease, but the liver was pale, large, and destitute of bile. One remarkable circumstance, is, that in many of the cases cited by M. Eisenmann, the oily evacuations had ceased, while the pancreas was so indurated as to render the performance of its functions impossible. The author also alludes to another circumstance, not less worthy of remark, viz., the large quantity of fat yielded, a quantity much greater than that contained in the food, and which leads to the supposition that part of the fœcal matters is transformed into fat. However that may be, the fact shows us that the presence of fat in the intestinal evacuations indicates with probability, but not with certainty, derangement in the functions of the pancreas; while, on the other hand, the absence of fatty matters does not authorise us to conclude that there is no disease in this gland, if other symptoms exist indicative of such an affection.

The author has endeavored to employ the facts collected by him in establishing a more correct symptomatology of disease of the pancreas. He divides the symptoms into two classes, those accompanying degeneration of the gland, and those arising from its acute or chronic inflammation. For the first of these, the symptoms are too various to afford any certain signs of the affection; the presence of fat in the evacuations, and the examination of the abdomen by the hand, suggest, but cannot positively determine, its presence. It is otherwise, according to the author, with inflammation of the pancreas. This is distinguished by a sense of weight in the epigastrium, which is especially manifest about two hours after eating, extending towards the breast with a feeling of oppression; loss of appetite, inodorous eructations, *malaise,* retching or even vomiting, although the tongue may remain clean; constipation; small, diminished pulse; emaciation; expression of features indicative of an abdominal affection; melancholy and sometimes weariness of life.

The author recommends, as a mode of treatment, the waters of Friedricks-hall, in small doses.—*Ed. Med. Jour. from Vierteljahrschrift fur die Praktische Heilkunde,* 1853.

